# A survival analysis in the assessment of the influence of the SARS-CoV-2 pandemic on the probability and intensity of decline in the value of stock indices

**DOI:** 10.1007/s40822-021-00172-7

**Published:** 2021-03-17

**Authors:** Beata Bieszk-Stolorz, Krzysztof Dmytrów

**Affiliations:** Institute of Economics and Finance, University of Szczecin, ul. Mickiewicza 64, 71-101 Szczecin, Poland

**Keywords:** SARS-CoV-2 pandemic, Stock indices, Survival analysis models, Risk assessment, C14, C41, G01

## Abstract

The aim of the study is to assess the strength of the world stock exchanges reaction to the SARS-CoV-2 coronavirus pandemic at the turn of 2019–2020. We analyze the risk and intensity of the decline in the values of the basic stock indices by means of selected methods of survival analysis. The spreading pandemic within a few months covered all continents and had a significant impact on the socio-economic situation of all countries. We studied the time of the 20% drop in stock market indices. This is a value that is taken as a sign of a crisis. In order to assess the probability of indices’ value decrease, we use the Kaplan–Meier’s estimator. We determine the risk of decline by means of a logit model and the intensity of the decline by means of an empirical hazard estimator and the Cox proportional hazard model. The intensity and risk of the decline of stock indices varied from continent to continent. The obtained results show that the intensity is highest in the fourth and eighth week after the peak and is the highest on European exchanges and then American and Asian exchanges (including Australia). The risk of falling the stock indices’ prices is the highest in America, followed by Europe, Asia and Australia, and lowest in Africa. Half of the analyzed indices record a 20% drop in value after 52 days (median duration). The study is a prelude to further analyses related to the crisis and the normalization of the situation on world stock exchanges. It allows to learn about the impact of the pandemic on the economic situation and to detect the differences between the continents.

## Introduction

In December 2019, a new strain of coronavirus (SARS‑CoV‑2) was discovered in Wuhan, the capital of the Chinese province of Hubei. It causes the novel coronavirus outbreak 2019 (COVID-19). On January 30, the outbreak was declared as a Public Health Emergency of International Concern (PHEIC) by the World Health Organization (WHO). On March 11th, the WHO declared that the virus was the cause of a global pandemic. As of June 7, 2020, the total number of infected people around the world is over 7 million and the death toll exceeded the 400 thousand mark. The SARS-CoV-2 is believed to be the ‘the once-in-a-century pathogen’ (the previous one was the Spanish influenza in the years 1918–1920) (Gates 2020). Since the declaration of the PHEIC, countries have begun introducing countermeasures, such as social-distancing, lockdowns and closing non-essential businesses. This caused the economic slowdown. Some of the restrictions were relaxed during the summertime. The full influence of the pandemic on the global economy is not yet known, because it continues to spread. Moreover, the second wave of the pandemic is expected during the autumn of 2020 and winter 2020–2021 and it is possible that the restrictions will be reinstated.

**Fig. 1:**
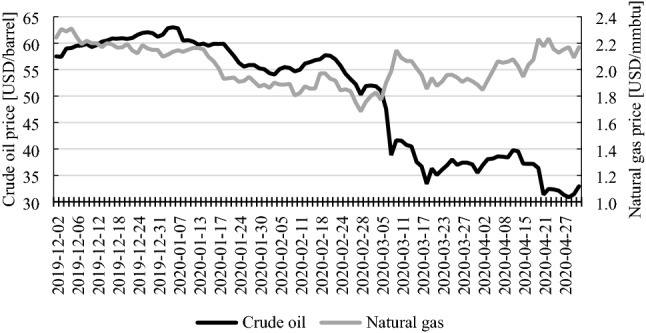
Crude oil and natural gas prices (December 2, 2019–April 30, 2020). Source: www.stooq.pl

The economic impact of the pandemic is visible in many areas, such as the decline in the values of stock indices, fall in prices of crude oil, gold, livestock, grains and other goods. They were observed after the declaration of the COVID-19 outbreak as a global pandemic. However, not all commodities noted a fall in their prices. Figure 1 presents the prices of crude oil and natural gas. It is clearly visible that the price of crude oil was decreasing since the beginning of 2020 and when the pandemic was declared, the price drop was much more rapid. Natural gas price, on the other hand, until the declaration of the pandemic had more or less the same course as the price of crude oil. After the global pandemic had been declared, its course was different—natural gas price started to increase.

**Fig. 2:**
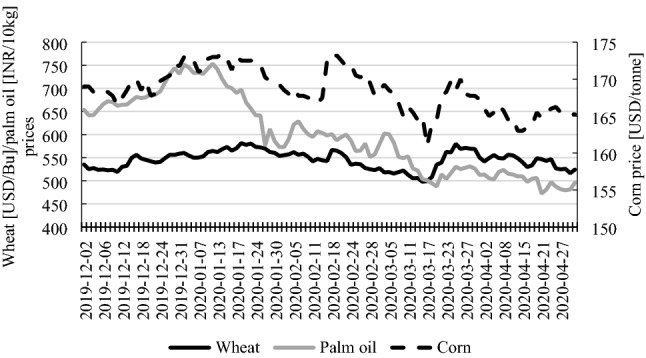
Wheat, palm oil and corn prices (December 2, 2019–April 30, 2020). Source: www.stooq.pl

Analysis of the prices of selected agricultural products (wheat, palm oil and corn) indicates that their variability is much higher than in the case of fuels. They had been decreasing during most of the period since the novel coronavirus was discovered (with the exception of corn price in mid-February). However, after the pandemic had been declared, their prices visibly increased, until late March and again began to decrease afterwards. This trend was observed until the end of the observation period (Fig. 2).

**Fig. 3:**
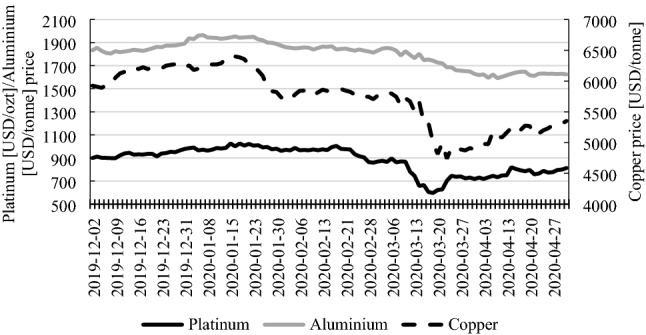
Aluminium, copper and platinum prices (December 2, 2019–April 30, 2020). Source: www.stooq.pl

The prices of selected metals (aluminium, copper and platinum) behaved in a quite different way than the above-mentioned ones (Fig. 3). The price of aluminium had been stable until the declaration of the pandemic and decreased afterwards. At the beginning of April, it stabilized on a new, about 10% lower level. The price of copper was characterized by much higher volatility. It had been increasing until mid-January and then dropped by about 10% by the beginning of February. Until the declaration of the pandemic, it had been stable and then dropped by almost 20% by the end of March. During the whole of April, until the end of the observation period, the price of copper was increasing. The price of platinum was stable since the beginning of the observation period (December 2, 2019), until the declaration of the pandemic. It then dropped by over 30% within 1 week. It had then regained over 20% within the subsequent week and was slowly increasing until the end of the observation period.

The aim of the study is to assess the strength of the global stock exchange reaction to the SARS-CoV-2 coronavirus pandemic at the turn of 2019–2020. We analyze the risk and intensity of the decline in the values of the basic stock indices. We use the non-parametric methods of a survival analysis in the study. We use the Kaplan–Meier’s estimator to assess the probability of indices’ value drop. We formulate the following research hypothesis:

**H1**: The risk and intensity of the fall in stock indices varies on a continental level.

## Literature review

The COVID-19 pandemic is a relatively new phenomenon. Its duration since the official date of the beginning of the outbreak—December 1, 2019—is just over 6 months and is ongoing. It is the main reason why the literature referring its economic impact is quite scarce, although many papers started to appear. Although it is obvious that this pandemic will have a great impact on the world economy, its strength, directions and range are difficult to assess because its nature is very different, from what we have faced in the past (for example the Spanish influenza pandemic in years 1918–1920, the Great Depression in the years 1929–1933, or the economic crisis of 2007–2009). The main challenges that lie in the present situation are (Fernandes 2020):

- it is a global pandemic,
- it is not focused on low-middle income countries,
- interest rates are historically low,
- the world is much more globalized,
- it is generating spill-over effects throughout supply chains,
- supply and demand are simultaneously devastated.

One of the most visual effects of the pandemic was the deep decline of the prices of many commodities—for example the price of a barrel of crude oil plunged from over 65 at the beginning of the year to less than 20 USD in mid-April—much lower than in the economic crisis of 2007–2009 (Ji et al. 2020). Also, prices of other commodities—agricultural products (like grains, edible oils, beverages, fertilizers), metals (copper, aluminium, and even precious metals, except for gold) noted a significant drop during the spread of the COVID-19 virus (World Bank 2020). Also, the news-based US index of Economic Policy Uncertainty (EPU) reached a level of over 400 points in April 2020 (Baker et al. 2020; Sharif et al. 2020)—the same was for the global economy. It is worth noting that during the 2007–2009 crisis its value did not exceed 170 points.

The second effect of the COVID-19 pandemic is the increase of the number of unemployed persons, thus a decrease of the employment rate. The most recent studies indicate that in the USA the employment ratio in February 2020 (just before the outbreak in the USA) was over 61%, while in the beginning of April 2020 it was 51%. The unemployment rate increased from 3.5% prior to the crisis to almost 11% in April 2020 (Coibion et al. 2020). Doerr and Gambacorta (2020) assess the situation on the European labor market in the aspect of employment risk indices. Their main findings are that the highest risk indices are for the regions, where the share of employment in small enterprises is high, as they depend heavily on local demand and are financially constrained. Therefore, the highest risk indices can be observed in Southern European countries (Italy, Greece, Slovenia, Croatia and in most provinces of France). The Baltic States, the Visegrad Group countries and Norway are characterized by medium risk indices, while Sweden, Germany, Denmark and the UK have low risk indices.

However, the first symptom of economic turbulences is visible the most on the changes of stock indices. When the spread of COVID-19 pandemic became apparent, most stock indices noted a sharp decline in their values. The US stock market reached the circuit breaker mechanism four times in 10 days, the main UK’s index, the FTSE, plunged more than 10% on March 12—the day after the global pandemic was declared (Zhang et al. 2020). The S&P 500 index noted a 30% decline in 16 trading days (Ali et al. 2020). Ashraf (2020) analyzes the impact of the number of confirmed COVID-19 cases and deaths on the main stock market indices in 64 countries by means of the panel data analysis. His main findings are that the reaction of the stock markets on the growth of confirmed COVID-19 cases is strong and negative, while the influence of the growth of confirmed deaths on the stock markets is also negative, but the strength of this relationship is much lower than in the former case.

Observation of the stock market during a crisis provides important information on the economic situation of a country or region. Pradhan (2018) has shown that there is a correlation between stock market development and economic growth per capita for the G20 countries using a time series from 1980 to 2015. The author of the study suggests that in order to ensure economic growth, attention should be paid to policies that promote the development of financial institutions and markets, including stock exchanges. Such actions may facilitate further investment and facilitate raising capital to support economic activity. The role of state policy in the process of recovery from the crisis is pointed out by Diemer and Vollmer (2015). They compare Japan with the Nordic countries of Norway, Finland and Sweden. They point out that the impact of similar policies on resolving financial crises in each of these countries in the 1990s is different. They also point out that long-lasting political processes, and especially long-term decision making, can make the crisis situation worse.

An interesting way of identifying the financial crisis of 2007–2009 is presented in the work by Olbryś and Majewska (2015). The procedure of determining the market conditions of Pagan and Sossunov (2003) is applied to determine the periods of crisis on the basis of an analysis of monthly logarithmic rates of return from the main indices of the Warsaw Stock Exchange—WIG and the New York Stock Exchange—S&P500. According to the definition of bullishness, during a series of quotations there must be a correspondingly large (at least 20%) increase/decrease in quotations, which means that the amplitude of the bullish phase is greater than or equal to 0.18, and the amplitude of the bearish phase is less than or equal to − 0.22. The identification of a crisis depends on its type. In their article Emin and Aytac (2016) present 20 different definitions of a currency crisis. They rely, among the others, on the research by Reinhart and Rogoff (2009), who assume that a currency crisis occurs when the value of a currency decreases by at least 15% during a year compared to the value of the dollar (or other currency accepted in a given country as a reference).

A survival analysis is used to analyze the effects of global crises. Research in this area refers to the phenomenon of the spread of the crisis on the economies of other countries and the analysis of the impact of the crisis on economic sectors in individual countries.

Puttachai et al. (2019) apply the Cox proportional hazard model to investigate the impact of the financial crisis in the US on the occurrence of crises in other countries. Out of 182 countries, 114 are not immune to the financial crisis in the US. The results indicate that countries in Africa, Asia and Australia, as well as those with higher economic development, have a lower risk of a crisis in 2008–2009. The high social development of the country increases this risk. This risk is not reduced by the country’s membership in the WTO and APEC. The authors postulate that the financial system of each country should be improved in order to stabilize the economy and protect it from the effects of the economic crisis from outside. Being a member of international trade organizations should, by definition, reduce the risk of a negative impact. If this is not the case, it means there is a “contagion effect”.

Okubo et al. (2014) analyze the impact of the global financial crisis of 2008 on Japanese exports in the machinery sector. The study uses data on Japanese trade (exports) at the product level. They analyze how the financial crisis has limited Japanese exports. Using the Cox regression model, they point out that trade in machinery parts and components to other Asian countries is less likely to be restricted due to the crisis. If this reduction takes place, it is more likely to revive. Regardless of the global financial crisis, Asian foreign trade is maintained, which has an impact on maintaining production networks in these countries. This is one of the main reasons for economic growth in Asia.

In his article, Evrensel (2008) applies non-parametric and parametric survival analysis methods to data on the crisis of international banks. Empirical results suggest that concentration in the banking sector is prolonging the survival time. He shows that the G-10 and non-G10 countries create two separate groups. The non-G10 countries have a higher frequency of failures, i.e. a higher risk of being affected by the banking crisis. Parametric regressions of survival time (Weibull) confirm the possibility that the impact of accompanying variables on banking crises may have different dynamics in the G-10 and outside the G-10. The results are consistent with the hypothesis of stability of concentration: concentration of banks reduces the risk of bank failure. Macroeconomic variables influence bank failure. The increase of inflation, money supply, national credit, and real interest rate increase the risk of bankruptcy. Moreover, the increase of real GDP, depreciation of national currency and improving trading conditions reduce this risk. Although bank restrictions, banking and economic freedom and responsibility reduce bank bankruptcy, high deposit insurance increases the risk of their bankruptcy.

Iwasaki and Kim (2018) use the Cox proportional hazard model to investigate the survival of more than 110,000 Russian companies in 2007–2015. They analyze the determinants of this survival during the global economic crisis, the subsequent internal Russian crisis in 2014–2015 and periods of economic growth. The number of board directors and auditors, the number of large shareholders, the age of the company and the network of contacts have a positive impact on the survival of the company during the recession. Companies with a closed type of legal form cope better during the crisis. Also, variables directly related to the company’s performance, such as ROA and gross margin, contribute significantly to the company’s survival. The impact of foreign ownership depends on the nature of the business cycle. At the time of the global financial crisis (2008–2009), such an effect proved negative, but became positive during the limited crisis in Russia (2014–2015). Similarly, it is recognized that freedom of management increases the risk of bankruptcy during the global financial crisis but decreased it during the recession in 2014–2015. The impact of state ownership is positive during normal periods, but not during recessions.

Bieszk-Stolorz and Markowicz (2017b) apply survival analysis methods to assess the volatility of the shares of companies listed on the Warsaw Stock Exchange during the bear market in 2011 and the following 2 years. They conduct the analysis using a logit model, Kaplan–Meier’s estimator and Cox regression model. They compare obtained results with previous results for the crisis period in 2008–2009. They examine 378 listed companies (listed in the whole analyzed period) and group them in three macro-sectors: industry, finance, and services. The research results confirm the hypothesis that the downturn in 2011 strongly affects the financial macro-sector. On the other hand, the hypothesis of a similar situation of macro-sectors during the financial crisis and the bear market is not confirmed for the financial and industrial sectors. The situation of the financial sector during the downturn is better than during the crisis, while for industry it is worse.

## Research methodology and statistical data

The study uses the survival analysis models. It is a set of methods aimed at studying the duration of phenomena occurring in various areas of human activity: in social, economic and political life. Originally, these methods were used in demographic research (duration of human life), medical research (recovery time) and technical research (failure-free time of machines and equipment). The methods of survival analysis are increasingly used to study the duration of socio-economic phenomena, including financial issues. The survival analysis methods are rarely used in the capital market analysis. However, more and more authors try to use them in this area (Lunde and Timmermann 2004; Deville and Riva 2007; Markovitch and Golder 2008; Bieszk-Stolorz and Markowicz 2017a, 2018).

The duration of a unit in a given state until a specific event terminating the observation occurs is assumed to be a random variable *T*. A study related to the application of survival models usually involves the observation of units belonging to a defined cohort. If the observation period is set, some of the units may not experience the event before its end and the duration is only partially known. Such observations are considered as right censored. A set of all complete and censored observations with survival times no shorter than *t* is referred to in the literature as a set of risk at time *t* and denoted by *R*(*t*) (Lee et al. 1975).

The basic concept is a duration function also called the survival function, defined as follows (Kleinbaum and Klein 2012):

1 $$S\left(t\right)=P\left(t>T\right)=1-F\left(t\right),$$

where *T*—duration, *F*(*T*)—cumulative distribution function of random variable *T*.

Using formula (1), duration quartiles can be determined. These are the moments of time for which the survival function takes the values 0.75, 0.5 and 0.25, respectively. The survival function determines the probability that a certain event will not occur until at least time *t*. Depending on the defined event, it is sometimes more convenient to analyze the cumulative distribution function *F*(*T*), expressing the probability that the event will not occur until at least time *t*. The most commonly used non-parametric estimator of the survival function is the Kaplan–Meier’s estimator defined by the formula (Kaplan and Meier 1958):

2 $$\widehat{S}\left({t}_{i}\right)=\prod_{j=1}^{i}\left(1-\frac{{d}_{j}}{{n}_{j}}\right) \quad {\text{ for }}i= 1,2 ,\dots ,k,$$

where *t*_*i*_—the point in time when at least one event occurs, ${t}_{1}<{t}_{2}<\cdots <{t}_{k},{t}_{0}=0$, *d*_*i*_—number of events in time *t*_*i*_, *n*_*i*_—number of units observed in time *t*_*i*_, ${n}_{i}={n}_{i-1}-{d}_{i-1}-{z}_{i-1}$, *z*_*i*_—number of censored observations in time *t*_*i*_.

We test the significance of differences between the two survival curves to compare the duration of the phenomena. We then verify the null hypothesis ${H}_{0}: {S}_{1}(t)={S}_{2}(t)$. Depending on the research problem, we can consider one of the following alternative hypotheses: ${H}_{1}: {S}_{1}(t)\ne {S}_{2}(t)$, ${H}_{1}: {S}_{1}(t)>{S}_{2}(t)$ or ${H}_{1}: {S}_{1}\left(t\right)<{S}_{2}(t)$. Despite a relatively large number of non-parametric tests to compare the survival function, there is no consistent group of criteria to decide which of the tests has the greatest power and should be used in the analyses. Studies carried out in Latta’s work (1981) indicate that the power of the tests varies depending on the size of the sample being tested, the censorship mechanism and the distribution of the density of the hazard function. In 1972, the Peto brothers proposed a modification of the Wilcoxon test based on Kaplan–Meier’s assessment of the survival function for combined samples (Peto and Peto 1972). This test is used when the hazard ratio between groups is not constant (Stevenson 2009). Although one of the assumptions of the Peto–Peto test is the equal distribution of censored observations in both groups, the advantage of this test is that it does not lose power in case of different censoring in groups. This test is used when we want to pay more attention to the initial parts of the survival curves.

The second important function in the survival analysis is the hazard function *h* describing the intensity of occurrence of an event at time *t*, provided that it survives to time *t*. It is defined as follows (Kleinbaum and Klein 2012, p. 34):

3 $$h\left(t\right)=\underset{\Delta t\to 0}{\mathrm{lim}}\frac{P\left(t\le T<t+\Delta t|T\ge t\right)}{\Delta t}.$$

To assess the intensity of the occurrence of an event in subsequent units of time, we use a formula for an empirical hazard. It is denoted by the following equation:

4 $${\widehat{h}}_{j}=\frac{{d}_{j}}{{n}_{j}{\tau }_{j}} \quad {\text{ for }}j= 1, 2,\dots ,k,$$

where *d*_*j*_—the number of events in the *j*-th time interval, *n*_*j*_—the number of units observed in the *j*-th time interval, *τ*_*j*_—the length of the *j*-th time interval.

The semiparametric Cox hazard model can be used to assess the relative hazard, which is defined by means of the formula (Cox 1972):

5 $$h\left(t,\mathbf{X}\right)={h}_{0}\left(t\right)\mathrm{exp}\left({\alpha }_{1}{X}_{1}+{\alpha }_{2}{X}_{2}+\cdots +{\alpha }_{n}{X}_{n}\right),$$

where $\mathbf{X}=\left({X}_{1},{X}_{2},\dots ,{X}_{n}\right)$—vector of independent variables, ${h}_{0}(t)$—baseline hazard, ${\alpha }_{1},{\alpha }_{2},\dots ,{\alpha }_{n}$—model coefficients, *t*—observation period.

This model allows assessing the intensity of the event at moment *t* in the selected group in relation to the reference group. We determine the hazard ratio by the formula *HR* = exp(*α*_*i*_).

To assess the chance/risk of a relative event, we use a logit model (Kleinbaum and Klein 2010):

6 $$\mathrm{logit}\left(p\right)=\mathrm{ln}\left(\frac{p}{1-p}\right)={\beta }_{0}+\sum_{i=1}^{n}{\beta }_{i}{X}_{i},$$

where $p=P\left(Y=1|{X}_{1},{X}_{2},\dots ,{X}_{n}\right)$—conditional probability of the occurrence of an event, *X*_1_, *X*_2_, …, *X*_*n*_—independent variables, $\beta$_1_, *β*_2_, …, *β*_*n*_—model coefficients.

The coefficients of the logit model allow determining the chance (odds/risk) of an event occurring at moment *t* in the selected group in relation to the reference group. Relative risk is determined by the formula *OR* = exp(*β*_*i*_).

Using the above-described models of the survival analysis, we examined 108 major stock exchange indices. They are listed in Table 5 in the Appendix. We observed their values from December 15, 2019 to April 15, 2020 and analyzed 20% decrease in the values of the indices. Due to the spread of the pandemic, we group them by continents. As only three indices are included in the case of Australia, we decide to add them to the Asian ones. Thus, four groups of indices have been distinguished: Europe, America, Asia + Australia and Africa. Not all the indices reached the threshold in the analyzed period. They are censored observations (20% of them). Table 1 shows in each group the percentage of indices which in the analyzed period recorded at least a 20% decline and those which did not have such a decline. Additionally, Table 1 presents the maximum and minimum time of decline in the indices. The first required falls occurred on the 19th day after the peak (Europe), and the last—on the 84th day (also Europe). The highest number of indices did not reach the 20% drop in Africa (44%).

**Table 1:** Structure of indices with respect to the achieved limit values and continents (*N* = 108). Source: Own elaboration

Groups	Full observation (%)	Censored observations (%)	Maximal duration (days)	Minimal duration (days)
Europe	84	16	84	19
America	87	13	75	20
Asia + Australia	82	18	80	20
Africa	56	44	73	29
Total	80	20	84	19

Full observation—observation that ended with a 20% decrease

Censored observation—observation that did not end with a 20% decrease

## Assessment of the probability, intensity and risk of the decline in the value of stock indices

We base the study on the Dow Theory (Rhea 1932), according to which a 20% decline in stock market prices is a determinant of the bearishness. Additionally, we assume that the phases of the stock exchange cycle (decline, increase, mid-range) should not be shorter than 4 months (Pagan and Sossunov 2003). For these reasons, the observation period of stock exchange indices lasted from mid-December 2019 to mid-April 2020 (4 months). The initial event was the moment when the index reached its maximum during the observation period, and the final event was the moment when the index recorded a 20% decrease in value compared to its maximal one. The random variable *T* is the time that elapsed between the initial event and the final event. If such a decrease does not occur, the observation is considered censored.

**Fig. 4:**
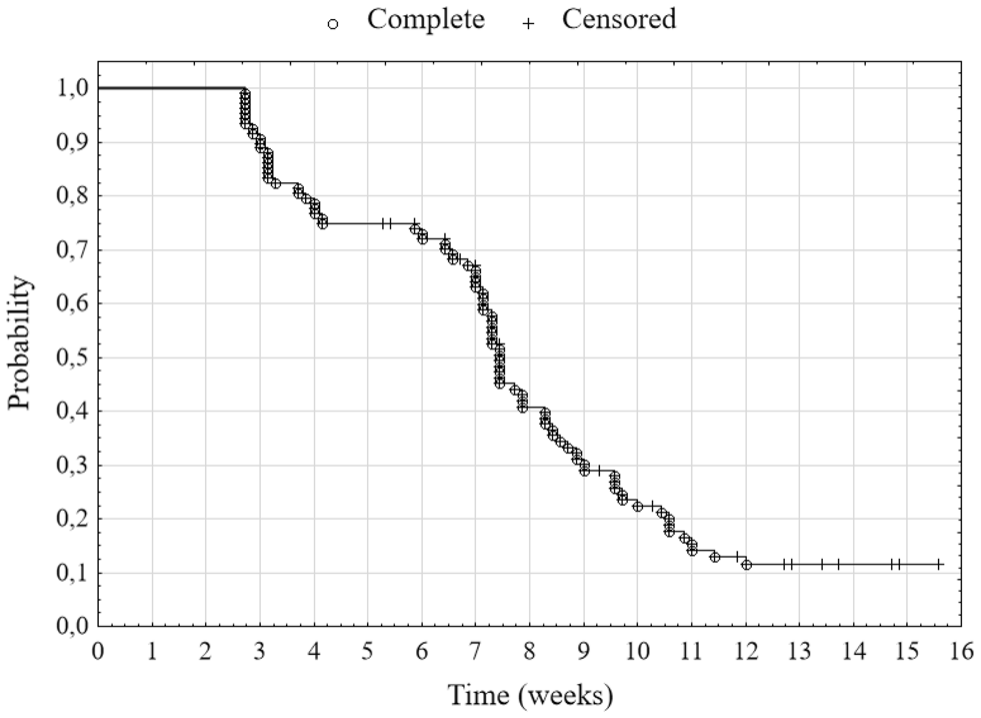
Kaplan–Meier’s estimator—probability of not reaching a 20% decrease in stock indices

**Fig. 5:**
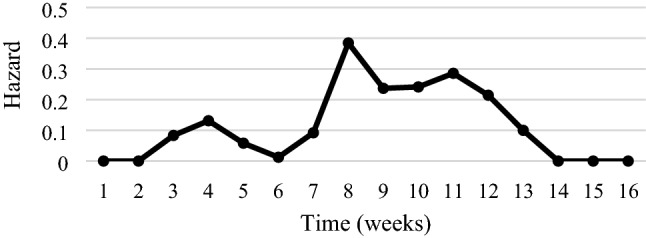
Intensity of a 20% decline in stock market indices—empirical estimator of hazard

In the first stage, using the Kaplan–Meier’s estimator, we assess the probability of not achieving a 20% decrease in the value of all indices together until time *t*. We observe the first such decreases starting from day 19 since the moment of reaching the maximum value. Half of the indices recorded a 20% decrease within 52 days after reaching their maximum value (median duration) (Fig. 4, Table 2). Subsequently, we determine the empirical hazard for all indices altogether. The Fig. 5 presents that the highest intensity of the decline in the indices took place in the fourth and eighth week. These are the two local maxima. It is also visible in the course of Kaplan–Meier’s estimator in Fig. 4. Between the third and fourth week and seventh and eighth week this estimator has significantly higher decreases in its value. It means that in these two periods the probability of a 20% decline in the values of the stock indices increased. In these periods also the largest numbers of indices lost 20% of their values.

**Table 2:** Quartiles of duration (*N* = 108)

Groups	First quartile (days)	Median (days)	Third quartile (days)
Europe	21	48	55
America	22	52	61
Asia + Australia	50	55	74
Africa	51	63	73
Total	29	52	68

**Fig. 6:**
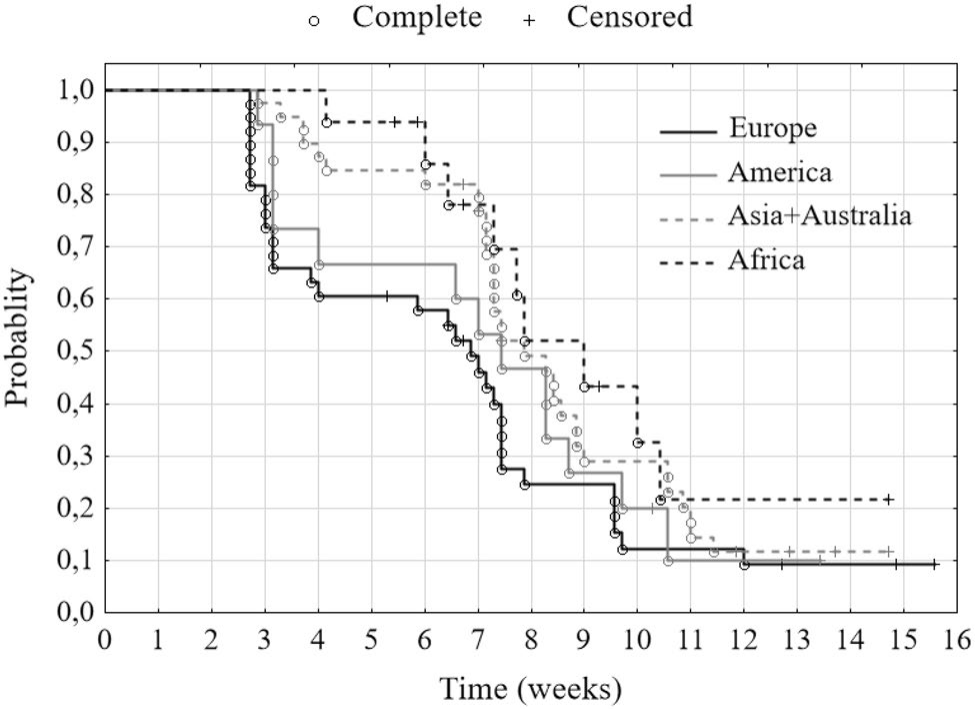
Kaplan–Meier’s estimator—probability of not reaching the 20% decrease in stock market indices by continent

In the second stage of the study, we calculated the Kaplan–Meier’s estimators for the index groups (Fig. 6), which are distinguished by their continental belonging. The European stock exchanges record the fastest 20% decrease in the indices, then the American, Asian and Australian ones, and the slowest—African ones. This is indicated both by the mutual position of the duration curves and the quartiles of duration (Table 2). The values of the first quartiles for Europe and America (21 and 22 days) show a faster and similar rate of decline of stock indices. However, in the case of Asia with Australia and Africa, the rate is also similar but much slower (50 and 51 days, respectively). In addition, we confirmed the existence of differences between the groups by a test for many groups (at a significance level of 0.01). A detailed examination with the Peto–Peto test for each pair of groups separately indicates that the null hypothesis about the equality of survival curves should be rejected (at a 0.01 significance level) for pairs: Europe and Asia + Australia and Europe and Africa (Table 3). The general conclusion that can be drawn from this is that the European stock exchanges noted the fastest 20% decline in their values in the whole analyzed period. The rate of decline was slightly slower in America and the slowest in Africa and Asia with Australia.

**Table 3:** Results of the Peto–Peto test (*N* = 108)

Groups	Test statistics	*p* value
Total	11.0897**	0.0113
Europe-America	1.1351	0.2563
Europe-Asia + Australia	2.6339***	0.0084
Europe-Africa	2.6008***	0.0093
America-Asia + Australia	1.1806	0.2377
America-Africa	1.4651	0.1429
Asia + Australia-Africa	0.7752	0.4382

The results of the Peto–Peto test are presented in the second column

**Significance level 0.05

***Significance level 0.01

In the third stage of the analysis, we assess the relative hazard and the risk of relative decline in indices on each continent. We determine this decline by the geographical location (continent) of the country (*x*_*i*_). This is a qualitative feature that has been converted into dummy (dichotomous) variables. The number of dummy variables must be one less than the number of categories. Stock exchange indices from African stock exchanges are used as a reference group (coded as 0). Earlier analysis show that this is the continent where the decline in indices is the slowest. There are therefore three variables in the models: *x*_1_ (Europe), *x*_2_ (America), *x*_3_ (Asia + Australia). The limit value for the decline is assumed to be 20%. In the case of the logit model, the dichotomous dependent variable *Y* takes the value of 1 if there is at least a 20% decline in the indices, and the value of 0 in the opposite case. We present the results of the estimation of the parameters of both models below in Table 4.

**Table 4:** Results of the Cox hazard model and the logit model parameters estimation (*N* = 108)

Variable	Parameter’s estimator	Standard error	Wald’s statistics	*p* value	Hazard/odds ratio
Cox hazard model					
* x*_1_	0.7563**	0.3779	4.0062	0.0453	2.13**
*x*_2_	0.5181	0.4341	1.4244	0.2327	1.68
*x*_3_	0.2946	0.3779	0.6077	0.4357	1.34
Logit model					
Intercept	0.2513	0.5040	0.2487	0.6180	–
*x*_1_	1.4227**	0.6722	4.4789	0.0343	4.15**
*x*_2_	1.6205*	0.9115	3.1604	0.0754	5.06*
*x*_3_	1.2685*	0.6543	3.7590	0.0525	3.56*

The second column presents estimators of parameters ${\alpha }_{i}$ for the Cox hazard model and ${\beta }_{i}$ for the logit model

Wald’s statistics in the fourth column is used for checking the statistical significance of regression coefficients for non-linear models

The last column presents the hazard ratio for the Cox hazard model and the odds ratio for the logit model

*Significance level 0.1

**Significance level 0.05

**Fig. 7:**
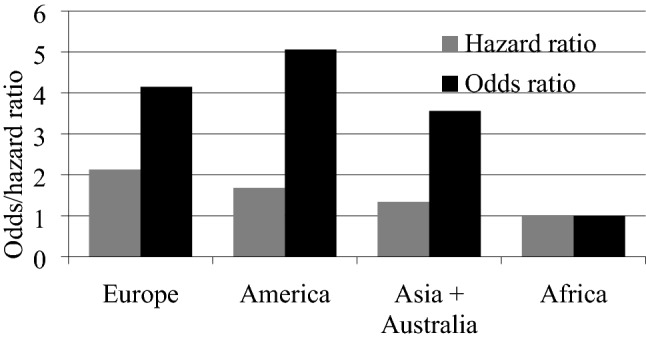
The risk and intensity of the decline of stock indices

For both models, we determine the hazard ratios (HR) and odds ratios (OR), respectively. These are shown on Fig. 7. Their value above 1 indicates a higher intensity or risk of decline than for the reference group (African continent). For both models all the hazard and odds ratios are greater than 1. For Africa alone, as a reference group, they are equal to 1. The decline intensity of the European stock exchange indices is highest and 2.13 times higher than for the African continent. The American indices have 1.68 times higher decline intensity than the African one and the Asian and Australian ones—1.34 times higher. The risk of declining stock exchange indices is greatest on the American exchanges and about 5 times greater than for African exchanges, for the European stock exchanges it is 4.15 times greater, and for the Asian and Australian ones—3.56 times greater. This means that the European and American markets reacted with the greatest strength to the pandemic situation.

## Conclusions

The analysis confirms that the response of global stock markets to the SARS-CoV-2 coronavirus pandemic is varied. The 20% drop in stock indices characterizing the crisis is noticeable in 80% of the analyzed stock exchanges. This shows that all stock exchanges reacted almost simultaneously to the spread of the virus. The analysis of the data indicates that individual stock exchanges differed with respect to the moment when the maximum value of indices was recorded, but the biggest drops took place in March 2020. It was after the WHO declared a state of pandemic (March 11, 2020). That is why there are differences in risk and intensity of falls observed in different countries and on different continents. The greatest risk of decline is on the American stock exchanges. This is due to the fact that as much as 87% of indices lost 20% of their value. On the other hand, European exchanges are characterized by the highest intensity of decline. It results from the fact that the time in which they declined from their maximal values was the shortest (median duration equal to 48 days). Also, a high percentage of them reached the required loss of 20% of their value. This can be explained by the fact that a large increase in the number of COVID-19 cases in Europe occurred earlier than on the American continent. During this time, the African indices noted the least decreases and the largest fraction of them did not recorded the required 20% decline. On the Asian continent the situation is ambiguous. In China and South Korea large increases in the coronavirus cases were observed at the beginning of the pandemic, while in Iran the increase occurred at a similar time as in European countries. In other Asian countries, e.g. India, the pandemic situation started to develop later. This confirms the research hypothesis. The article is an introduction to further research. Interesting is the chance and intensity of the increase in the value of indices to pre-crisis levels and the impact of the second wave of the disease on the stock market, which is expected in the autumn of 2020.

## Appendix

See Table 5.

**Table 5:** Stock indices used in the study

Country	Index	Country	Index	Country	Index
Argentina	MERVAL	Ireland	ISEQ	Russia	MOEX
Australia	S&P/ASX200	Israel	TA35	Russia	RTS
Austria	ATX	Italy	FTSEMIB	Rwanda	ALSIRW
Bahrain	Bahrain All Share	Ivory Coast	BRVM10	Saudi Arabia	MSCI TADAWUL 30
Bangladesh	DSE30	Jamaica	JSEMI	Saudi Arabia	TASI
Belgium	BEL20	Japan	NIKKEI	Serbia	BELEX15
Bosnia and Herzegovina	BIRS1	Jordan	AMGNRLX	Singapore	STI
Bosnia and Herzegovina	SARAJEVO10	Kazakhstan	KASE	Slovakia	SAX
Botswana	BSE	Kenya	NSE20	Slovenia	SBITOP
Brazil	BOVESPA	Lebanon	BLSI	South Korea	KOSPI
Bulgaria	BSE SOFIX	Malaysia	KLCI	South Korea	KS50
Canada	TSX	Malta	MSE	Spain	IBEX35
Chile	SASEIPSA	Mauritius	MDEX	Sri Lanka	CSE
China	SSECOMP	Mexico	BMV	Sweden	OMXS30
China	SZSECOMP	Mexico	FTFTBIVA	Switzerland	SSMI
Columbia	COLCAP	Mongolia	MNETOP20	Thailand	SETI
Croatia	CROBEX	Montenegro	MNSE10	Taiwan	GTSM50
Cyprus	Cyprus Main Market	Morocco	MASI	Taiwan	TAIEX
Czechia	PX	Namibia	FTN098	Tanzania	DSEI
Denmark	OMXC20	Netherlands	AEX	Tunisia	TUNINDEX
Ecuador	Guayaquil Select	New Zealand	NZMC	Tunisia	TUNINDEX20
Egypt	EGX30	New Zealand	NZX50	Turkey	BIST100
Egypt	EGX70	Nigeria	NGSE30	UAE	ADXGENERAL
Finland	OMXH25	Norway	OBX	UAE	DFMGI
France	CAC40	Norway	OSEBX	Uganda	ALSIUG
Germany	DAX	Oman	MSI	Ukraine	PFTSI
Germany	STOXX50E	Pakistan	KSE	United Kingdom	FTSE
Greece	THEXCOMP	Peru	SPBLPGPT	USA	DJI
Hong Kong	FTXIN25	Philippines	PSEICOMP	USA	NASDAQ
Hong Kong	HANGSENG	PHNA	PLE	USA	NDX
Hungary	BUX	Poland	WIG20	USA	SP500
Iceland	OMXIPI	Poland	WIG30	Venezuela	IBC
India	NSEI	Portugal	PSI20	Vietnam	HNX30
India	SENSEX	Qatar	QSI	Vietnam	VNI
Indonesia	IDXCOMP	Romania	BETI	Vietnam	VNI30
Iraq	ISX60	RSA	JTOPI	Zambia	LASILZ
